# Development and validation of a psoriasis treatment acceptability measure through group concept mapping

**DOI:** 10.1186/s12955-023-02162-6

**Published:** 2023-08-08

**Authors:** Stacie Hudgens, Amy Howerter, Shannon Keith, Colby Evans, Corey Pelletier

**Affiliations:** 1 Clinical Outcomes Solutions, 1820 E River Rd, Ste 220, Tucson, AZ 85718 USA; 2 Clinical Outcomes Solutions, Chicago, IL USA; 3 Evans Dermatology, Austin, TX USA; 4 Celgene Corporation (Former), Summit, NJ USA

**Keywords:** Concept elicitation, Group concept mapping, GCM, Patient-reported, Psoriasis, Psychometric, Patient preference, Treatment acceptability questionnaire, TAQ

## Abstract

**Background:**

Psoriasis is a common autoimmune dermatologic condition which has a pronounced negative impact on patient quality of life and disease burden. Currently, there are a number of treatments available for psoriasis, with differences in efficacy, mechanism of action, mode of administration, adverse effects, and tolerability. However, a reliable, validated patient-reported instrument to address patient expectations and of psoriasis treatment has not been developed. This project was undertaken with the aim of developing a fit-for-purpose self-reported instrument to inform patient expectations and preferences of psoriasis treatments.

**Methods:**

Two studies, both utilizing qualitative and quantitative methods, were conducted in patients within the entire spectrum of psoriasis severity. In Study 1, a group concept mapping (GCM) exercise was conducted with dermatologists and moderate-to-severe psoriasis patients to identify concepts important in the treatment of psoriasis. In Study 2, a preliminary Treatment Acceptability Questionnaire (TAQ) was developed using GCM-derived concepts from Studies 1 and 2, followed by cognitive debriefing (CD) telephone interviews of the preliminary TAQ. In Study 2, another GCM exercise was conducted with mild and newly diagnosed psoriasis patients. Psychometric analyses were performed on the TAQ to evaluate validity and reliability.

**Results:**

The Study 1 GCM exercise generated 43 concepts from moderate-to-severe psoriasis patients (n = 20) and dermatologists (n = 10). In Study 2, 37 GCM concepts were generated from mild and newly diagnosed psoriasis patients (n = 20). From the 2 GCM exercises, 28 concepts were selected to form the preliminary TAQ; CD interviews indicated strong understanding and relevance of TAQ items among patients with disease ranging from mild to severe. The final TAQ consisted of 20 items; psychometric analysis demonstrated strong validity and reliability of the TAQ.

**Conclusions:**

The TAQ is a novel psychometrically validated patient-reported instrument to inform healthcare providers of patients’ expectations of and preferences for treatment of their psoriasis and can help in shared decision making between patients and physicians.

**Supplementary Information:**

The online version contains supplementary material available at 10.1186/s12955-023-02162-6.

## Background

Psoriasis is among the most prevalent autoimmune diseases in the United States, affecting up to 3.0% of the adult population [1]. It is a chronic, symptomatic, immune-mediated systemic and skin condition characterized cutaneously by erythematous, scaly papules, and plaques resulting from rapid hyperproliferation of the epidermis [2]. Psoriasis is a recurrent condition with cyclic flares that can last from weeks to months [3].

The symptoms and treatment of psoriasis have a pronounced negative impact on patient-reported quality of life (QoL) [4]. Survey data collected from more than 5000 patients by the National Psoriasis Foundation between 2003 and 2011 show that psoriasis causes significant impairment of QoL and work productivity [5]. Further, psoriasis is a systemic disease associated with increased risk of several comorbidities, such as cardiovascular disease, Crohn’s disease, uveitis, and type 2 diabetes [6–8] as well as an increased risk of psychological complications such as depression, anxiety, and suicidality [9]. In addition, psoriasis causes a substantial economic burden [10, 11].

Multiple studies have revealed unmet needs as well as considerable patient dissatisfaction with current psoriasis treatments [12–18]. Commonly used patient-reported instruments such as the Psoriasis Index Quality of Life (PSORIQoL), [19] the Psoriasis Disability Index, [20] and the Psoriasis Symptom Inventory [21], and the Patient Benefit Index (PBI) [22] inform disease burden and impact of psoriasis and treatment outcomes, however, they do not provide the patient’s perspective on how to abrogate unmet needs of treatment. A survey of 18 validated PRO instruments found that the focus on patient QoL may not accurately capture patient treatment priorities (e.g., clearance of psoriasis) which can tie to reimbursement from payors [23]. Further, some instruments were reported to have limited psychometric validity [24, 25].

The present two-part mixed-methods study was conducted on patients and clinicians using group concept mapping (GCM), qualitative interviews, and quantitative data collection and analysis. The GCM technique is an alternative method to existing extensive interviewing and code-based qualitative analysis [26] and in this study was utilized to elicit concepts from both patient and clinicians about their expectations of an ideal treatment of psoriasis. The GCM methodology has been used in various research fields, including healthcare to enable patient responses and critical ratings of concept importance [27–31]. The goal of the GCM process is to gather concepts generated by and categorized by patients and clinicians pertaining to impacts of treatment, with little interference from or interaction with the researchers. First, participants generate responses to a prompt question, then they sort the responses into categories, and lastly they rate all of the responses in relation to specific questions. Quantitative analysis of the sorting and rating results, using multidimensional scaling and hierarchical cluster analysis, produces a shared framework that is informed by both patients and clinicians. Using this framework, an instrument was created and tested for psychometric reliability and validity with a cognitive debriefing interview. The ultimate aim of this project was to gather patients’ and clinicians’ responses to develop a validated instrument to inform patient expectations of an ideal treatment of psoriasis.

## Methods

### Overall study design

**Fig. 1:**
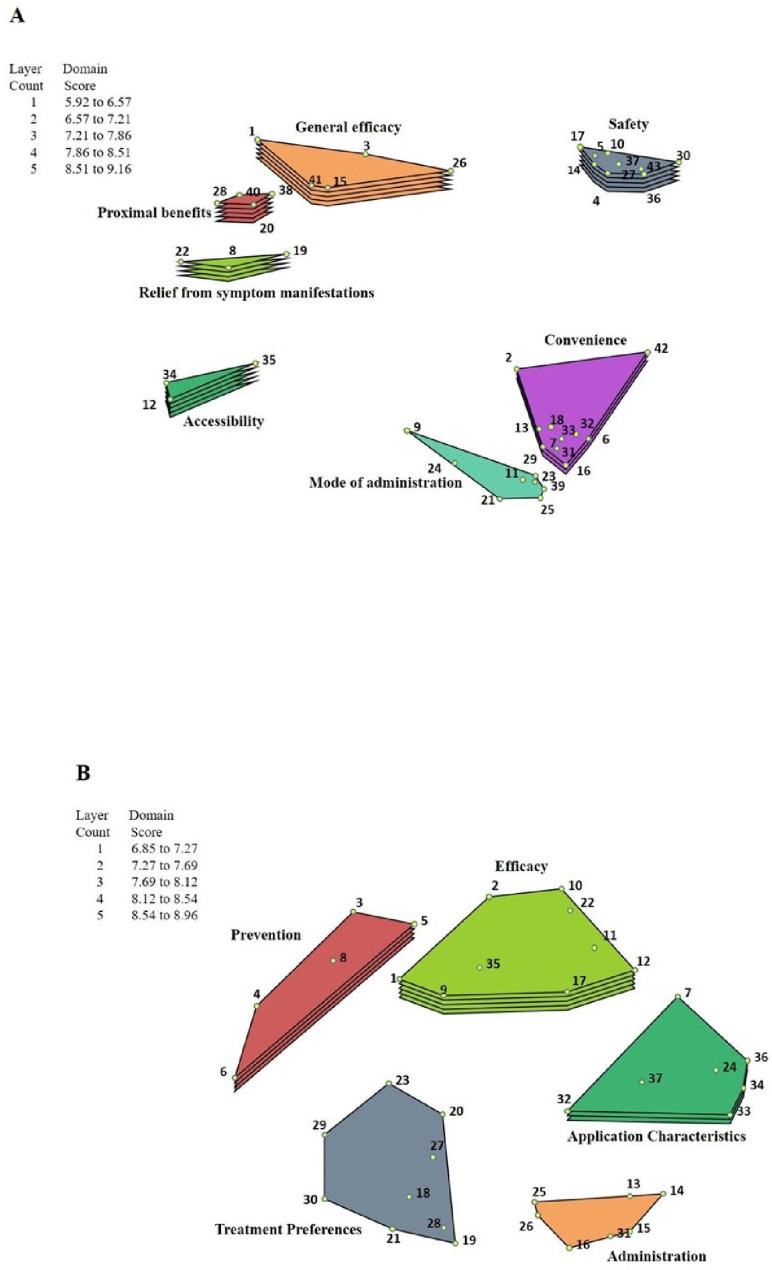
Study 1 Cluster Rating Map of GCM for **(A)** Study 1 (moderate-to-severe patients) and **(B)** Study 2 (mild patients). Combined ratings from dermatologists and patients of the relative importance of each concept were averaged by domain to deduce an average value for each domain on the 0‒10 scale. The lowest and highest domain averages were calculated, and the range between these two average scores was then “sliced” into quintiles and a rating map generated. The number of layers corresponds to stratified domain scores as shown by Layer Count vs. Domain Score; thus, more layers correspond to a higher range of importance ratings. The numbers in the polygons are the item numbers listed in Table 1

**Fig. 2:**
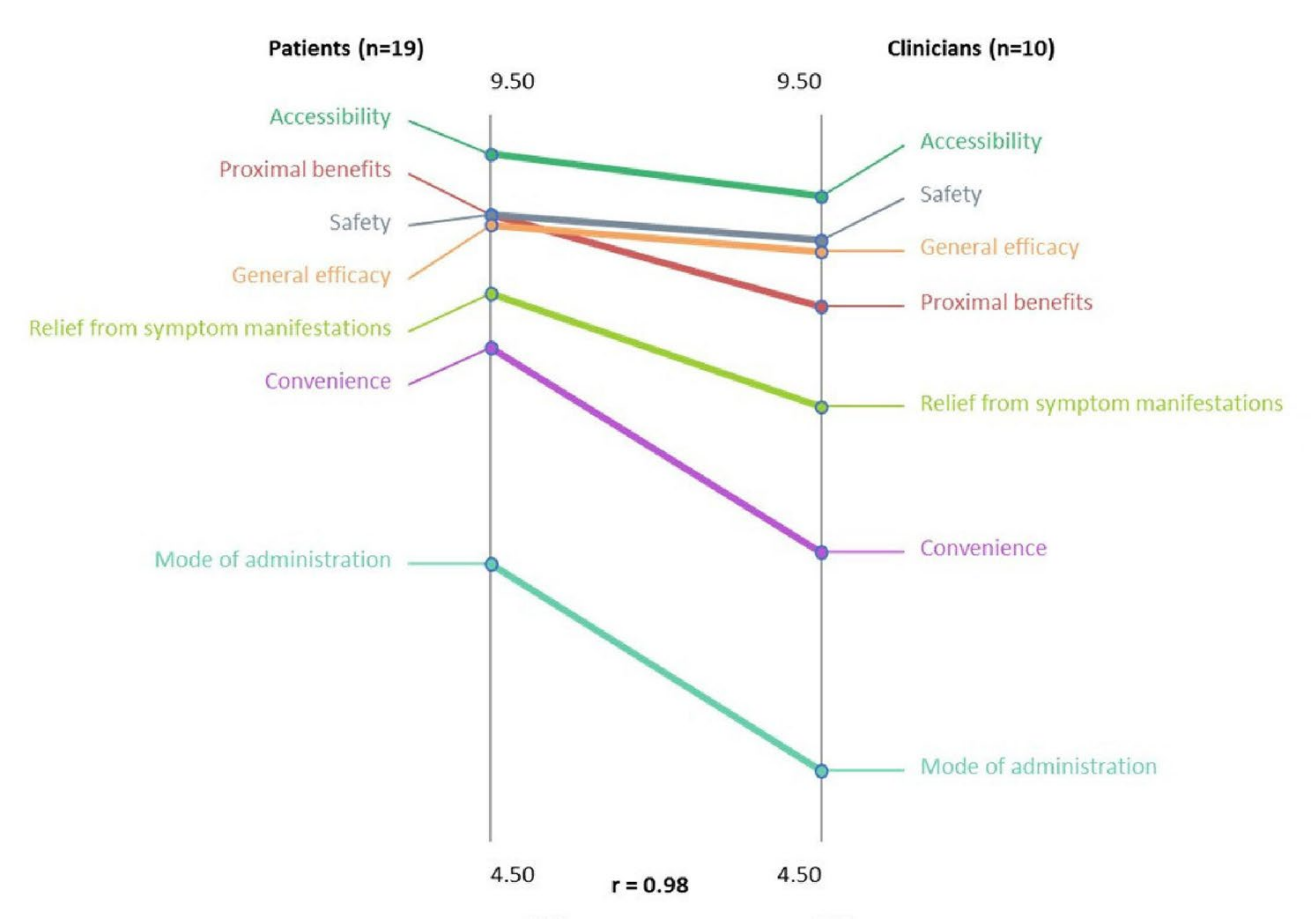
Pattern Matching of Domains Between Patients and Clinicians. Domain values were plotted on 2 parallel vertical axes based on patient and clinician ratings respectively, and straight lines were drawn to connect the same domains between the vertical axes. A horizontal line connecting the same domains on the vertical axes indicated perfect correlation, whereas sloping lines indicated various degrees of partial correlation

This investigation consisted of 2 studies to capture patient perspectives across the psoriasis severity spectrum in one comprehensive instrument. In Study 1, a GCM [32] exercise was conducted with patients with moderate-to-severe psoriasis, and dermatologists experienced in treating psoriasis to identify concepts important in the treatment of psoriasis. The GCM methodology combines qualitative and quantitative processes; steps in the GCM exercise are presented below. In Study 2, an additional GCM exercise to inquire from mild psoriasis patients, including some newly diagnosed patients, was conducted to complement the GCM exercise from Study 1, which was only fielded in moderate-to-severe psoriasis patients. Based on findings from both GCM exercises, a de novo Treatment Acceptability Questionnaire (TAQ) was generated. Next, cognitive debriefing (CD) interviews with a new sample of patients (spanning mild to severe psoriasis) were conducted to evaluate the content validity of the questionnaire. Each of these procedures is described in detail in the following sections. A GCM schematic (Fig. 1S) as well as a flowchart of the 2 studies (Fig. 2S) are provided in Appendix 1. The sample size of 20 participants per study is within the recommended guidelines for GCM studies [33], and the sample size of 200 for the online survey and 20 for the cognitive debriefing interviews (Study 2) is within recommended guidelines for analysis [34].

**Table 1:** Sorting of the Harmonized Concepts Generated in GCM Exercises

Domain	Study 1 GCM (Moderate–Severe Patients): 43 Harmonized Concepts Generated	Study 2 GCM (Newly Diagnosed–Mild Patients): 37 Harmonized Concepts Generated
Proximal Benefits	**20. Clear outside symptoms, such as dryness and redness**	—
	28. Be effective in treating joint disease	—
	38. Help control the itching	—
	**40. Be effective in treating skin disease, including nails and scalp**	—
Relief from Symptom Manifestations	8. Free a patient from hiding problem skin	—
	19. Consider how much the symptoms affect a patient’s daily life	—
	22. Free a patient from feeling embarrassed	—
Efficacy/General Efficacy	1. Resist building a tolerance	1. Result in the disappearance of rash
	**3. Not lose efficacy over time**	2. Relieve the itching and associated discomfort
	15. Be predictably effective	7. Absorb invisibly in my skin
	26. Be strong enough to be effective, but mild enough not to cause other sicknesses or symptoms	9. Be effective with little to no side effects
	41. Be effective	10. Control the itching
	—	11. Work fast and effective for thicker plaques
	—	12. Work longer than two hours
	—	17. Safe for long term daily use
	—	22. Control the itch longer
	—	35. Have little to no side effects
Accessibility	12. Be affordable under a patient’s insurance plan	—
	34. Be available under a patient’s insurance plan	—
	35. Not interfere with a patient’s occupational responsibilities	—
Safety	4. Be safe for long term use (patient-generated)*	—
	5. Not damage a patient’s liver	—
	10. Not increase the risk of infection, cancer, or other disease	—
	14. Be safe to use for those with pre-existing conditions, such as renal failure, liver disease, or internal malignancy	—
	17. Be safe for long-term use (clinician-generated)*	—
	27. Not lower a patient’s immune system	—
	30. Be safe to use during pregnancy and lactation	—
	36. Have minimal, non-life-threatening side effects	—
	37. Consider potential side effects	—
	**43. Have minimal interactions with other drugs and medications**	—
Convenience	2. Be able to adjust dosage to psoriasis severity	—
	**6. Be simple and quick to administer**	—
	**7. Be able to take with or without a meal**	—
	13. Not be very time-intensive or consuming	—
	**16. Have an alternative to injections**	—
	**18. Be easy to include in a patient’s normal routine**	—
	31. Not be messy	—
	32. Be convenient and easily managed by a patient	—
	33. Be easy to administer	—
	**42. Be painless to use**	—
Administration/Mode of Administration	9. Not require multiple drugs	13. Require less frequent applications throughout the day
	11. Be a medication implanted just below the skin	14. Is effective using once to twice weekly
	21. Be an oral medication taken once a day	15. Be a once a day application that lasts
	23. Be a topical ointment or cream	16. Be a once a month injection
	24. Not require lab monitoring	25. Be easy to apply to all areas which need treatment
	25. Be a once monthly injection	26. Come with gloves for application
	29. Have infrequent dosing	31. Be a pill for long term use
	39. Be an oral medication	—
Application Characteristics	—	7. Absorb invisibly in my skin
	—	24. Not affect the way my hair looks
	—	32. Not feel oily on the skin
	—	33. Be gentle enough for daily use on my scalp
	—	36. Not leave my hair oily
	—	37. Not damage clothing
Treatment Preferences	—	18. Be a non-steroidal cream
	—	19. Have a hands free applicator
	—	20. Double as a safe mechanism for scratching
	—	21. Be easy to apply to the scalp
	—	23. Not be greasy
	—	27. Be an exfoliating solution that can get rid of the scale and flakes
	—	28. Be a liquid to give immediate relief
	—	29. Be gentle enough to use daily without damaging my hair
	—	30. Not involve placing oily drops in my ears
Prevention	—	8. Clear the skin and keep it clear
	—	3. Prevent symptoms from occurring
	—	4. Prevent plaque buildup on my scalp
	—	5. Work from the inside to prevent itching
	—	6. Start with managing the triggers, like stress

*This concept was inadvertently retained as a duplicate. Concept numbers were assigned by the GlobalMax software after sorting and rating and are not ordered by domain category, pharmacological characteristics or alphabet. The 10 concepts bolded were selected for online survey as described later

### Participant recruitment

Patients for both studies were recruited from collaborating clinical facilities using a recruitment agency. Recruitment took place in Aug – Oct 2017 (Study 1) and Oct 2018 – Sep 2019 (Study 2). In Study 1, patient participants had to be aged > 18 years and have a clinician-confirmed diagnosis of current moderate/severe psoriasis, have proficiency in written and spoken English, and internet access. Patients were excluded if a mental or physical condition prevented participation; were currently enrolled in other psoriasis interventional or QoL studies; or had a history of alcohol or substance abuse in the past 12 months. Clinician participants were practicing dermatologists in the US. For Study 2, only patients with mild psoriasis, including newly diagnosed patients, participated in the GCM exercise. Additional patients who participated in the CD interviews in Study 2 represented the full range of disease severity. For all patients in Study 1 and 2, although severity was reported both by patients and dermatologists, the selection of patients in these studies was based on dermatologist reported severity using a Clinician Global Impression – Severity (CGI-S) rating. All participants were compensated at $75–$300 with the amount depending on which study they participated in and whether they were patients or clinicians.

### Study 1 and 2 GCM procedures

The GCM exercise consisted of multiple steps (shown in Fig. 1S of the Supplement). At the outset, participating patients and dermatologists used the GlobalMAX software (Concept Systems Inc., Ithaca, NY, USA; Task 1) and completed a concept elicitation exercise in response to an online prompt: “An ideal treatment to manage the symptoms of psoriasis should…”[*for patients ‘my’ was substituted for ‘the’*]. Participants were instructed to type in as many individual responses to the prompt as they wished and were able to view responses from earlier participants. All concepts were examined and harmonized by the research team in a process that involved removing redundant responses and spelling corrections to create a final list of concepts. After the research team determined the final list of concepts (which took approximately 2 to 3 weeks), the same participants were asked to return to the GCM platform and sort all concepts into groups of concepts (domains) that the participants considered were conceptually related (Task 2). Directly after the sorting exercise, the same participants were prompted within the software to rate all concepts based on “how important is this to you in terms of overall treatment for psoriasis?” on an ascending scale of 0 to 10, with 0 representing “not at all important” and 10 representing “extremely important.” This combination of sorting and rating activities performed by the participants provides the patient’s own perspective on relevance and importance as well as input from clinicians to examine to what extent clinicians’ and patients’ perspectives overlap and was used for the GCM analysis.

### GCM analysis

A 2-dimensional (2D) point map was generated to visually represent the relationships between the concepts identified in Task 1. Sorting data from Task 2 were incorporated into a similarity matrix that calculated the frequency with which participants grouped each concept with another concept; nonmetric multidimensional scaling was then used to generate coordinates for each concept, which were plotted to create a 2D relational distribution of concepts. Fit statistics of the map were assessed to ensure adequate fit [35]. The goodness-of-fit, determined by the distance of values in the input similarity matrix, was measured by the stress value; it is estimated that a stress value range between 0.205 and 0.365 will be yielded in approximately 95% of concept mapping projects [36] and considered a good fit. A lower stress value implies a better fit [36]. The concept-level coordinates were subjected to hierarchical cluster analysis of group-related concepts, which were used to create the cluster map. A polygonal shape was configured for each cluster map, called a domain, which encompassed related concepts as specified by participants. Combined ratings from dermatologists and patients of the relative importance of each concept were averaged by domain to deduce an average value for each domain on the 0‒10 scale. The lowest and highest domain averages were calculated, and the range between these 2 average scores was then “sliced” into quintiles and a rating map generated. In Study 1, pattern matching diagrams were generated to compare average domain ratings between patients and dermatologists. Domain values were plotted on 2 parallel vertical axes, one axis based on patient ratings, the other on dermatologist ratings, and straight lines were drawn to connect the same domains between the vertical axes. A horizontal line connecting the same domains on the vertical axes indicated perfect correlation, whereas sloping lines indicated various degrees of partial correlation.

Selection of final concepts from the GCM exercise for a potential patient-reported measure requires careful examination of all point-maps, domain clustering of concepts, ratings distribution, and pattern-matching diagrams across all of the domains by the research team. The purpose is to select a list of concepts that best represents all of the unique concepts generated by the sample. For example, the final list of concepts included at least one from each domain to ensure concept coverage. Additionally, the concepts rated highest in importance were reviewed for inclusion. For Study 1, 10 concepts were selected across the domains. For Study 2, consideration of the Study 1 concepts, in addition to the patient-generated concepts from the Study 2 GCM exercise, was used to compile the concepts for a preliminary TAQ that was included in the Study 2 CD interviews.

### Study 2 cognitive debriefing

In Study 2, CD telephone interviews involved debriefing each item and asking questions regarding the participants’ overall impression of the questionnaire’s usefulness in aiding communication with their clinician. Newly recruited participants who did not participate in the GCM of all psoriasis severity levels were interviewed by telephone and audio-recorded on the preliminary 28-item TAQ which represented selected concepts generated from both GCM exercises. Participants were emailed a copy of the measure one day before their interview. In the first phase of the CD interview, participants read out loud the instructions, items, and all response options, then verbally described their reasoning for selecting a particular response. Participants were next asked to put each item into their own words, what they were thinking about when they selected an answer, whether the item was relevant to their experience of psoriasis, and whether they had any suggestions for changing the item. Once all items were debriefed, participants were asked to compare 2 rating scales, a 4-point scale and an 11-point scale, and whether they preferred one over the other and why.

For the second phase of the CD interview, comprising general questions regarding the usability of the questionnaire, participants were asked whether they consider that the questionnaire would aid in their communication with physicians about their treatment preferences and whether they would be comfortable sharing their answers with their doctor. Based on the previous GCM results and the CD interview results, a final list of concepts was selected to proceed with the final version of the TAQ.

### Study 2 online survey

Following the CD interviews, concepts comprising the final version of the TAQ were programmed into an online survey utilizing Qualtrics online survey platform [37]. In addition the Dermatology Life Quality Index (DLQI) was included for validity comparisons [38]. A new sample of psoriasis patients, who had neither participated in the GCM or CD interviews, across the range of severities completed the survey. If a patient did not complete the survey within 3 days, a reminder was sent. Patients received up to 3 reminders before being considered lost to follow-up and were replaced with additional subjects.

### Psychometric analysis

Psychometric analyses were performed on the final TAQ data obtained from the Study 2 online survey.

### Individual item performance

Descriptive item characteristics were provided for each item on the TAQ. The frequency and percentage of each response option were presented for the overall study and by severity group. Floor and ceiling effects were displayed as the proportion of participants who respond at the lowest response option and the highest response option, respectively, for an item. Since there were 4 possible response options on the TAQ, a threshold of 25% was set to define floor and ceiling effects.

### Item correlation

The relationship between items was explored with inter-item correlations, where items with high correlations (typically > 0.80) could indicate potential item redundancy [39]. Item-total correlations were examined to test whether any item is inconsistent with the average of the other items (total score on TAQ) using the threshold of < 0.4 [39].

### Dimensionality and structure

Two techniques were used to identify the dimensionality and structure of the TAQ; Exploratory factor analysis (EFA) was used to assess the latent (underlying) factors or dimensions within the observed data. Confirmatory factor analysis (CFA) was also used to evaluate how well the study data fit the GCM proposed structure.

### Internal consistency

Homogeneity of the TAQ was estimated by calculating Cronbach’s alpha coefficient [40]. Values greater than 0.70 are indicative of acceptable internal consistency among item scores [41].

### Known-groups validity

Known-groups validity examines the ability of a measure to discriminate between different populations where a difference between populations is expected. The total TAQ score was compared between online survey participants based on groups defined by the DLQI and CGI-S. For the DLQI, participants were categorized into the following groups based on their DLQI total score and effect on patient’s life: 0–1 = no effect at all; 2–5 = small effect; 6–10 = moderate effect; 11–20 = very large effect; 21–30 = extremely large effect. The CGI-S rates psoriasis severity on a scale of 1 to 5: 1 = none; 2 = mild; 3 = moderate; 4 = severe; 5 = very severe. An ANOVA was used to test statistically for the differences in TAQ scores between the DLQI groups and CGI-S severity groups. If significant differences are found between groups, known-groups validity is supported.

## Results

### Patient demographics

For each study, the majority of participants were female, non-Hispanic, White (except for Study 2 CD interview and online survey, which had a majority African-American participants), and group mean ages ranged from 39 to 51 years. Participants in Study 1 GCM (N = 20) had primarily moderate (75%) psoriasis, as reported by dermatologists. For the Study 2 GCM phase (N = 20), all participants (100%) had mild/very mild disease severity. Participants in Study 2 CD interview and online survey phases were generally evenly distributed across disease severities (Table 2**)**. Of the 200 recruited for the online survey, 198 returned responses (99% response).

**Table 2:** Patient Demographics and Disease Severity

	Study 1: GCM (N = 20)	Study 2: GCM (N = 20)	Study 2: CD Interview (N = 20)	Study 2: Online Survey (N = 198)
Sex, n (%)				
Female	11 (55)	16 (80)	15 (75)	111 (56)
Male	9 (45)	4 (20)	5 (25)	87 (44)
Age, years				
Mean (SD)	50.6 (14.4)	45.6 (18.4)	51.1 (19.0)	39.3 (12.7)
Min/Max	28/85	25/84	19/80	18/78
Race, n (%)				
White/Caucasian	17 (85)	15 (75)	5 (25)	77 (39)
Black/African-American	3 (15)	5 (25)	11 (55)	99 (50)
Asian/Asian-American	0	0	1 (5)	2 (1)
Other	0	0	3 (15)	20 (10)
Ethnicity, n (%)*				
Hispanic/Latino	0	0	1 (5)	21 (11)
Not Hispanic/Latino	20 (100)	20 (100)	18 (90)	177 (89)
Disease Severity, Clinician-rated, n (%)				
None/Very mild	-	-	-	1 (< 1%)
Mild	0	20 (100)	5 (25)	63 (32)
Moderate	15 (75)	0	5 (25)	74 (37)
Severe	5 (25)	0	4 (20)	46 (23)
Very Severe	0	0	6 (30)	14 (7)

*1 missing in Study 2: CD Interview

### Clinician background

Ten dermatologists completed the GCM exercise in Study 1. Two had been in practice for 1 to 5 years, one had been in practice for 5 to 10 years, and seven had been in practice more than 10 years. The majority of dermatologists reported being in private practice and three reported being in academic practice. They reported seeing patients specifically for psoriasis from 0 to 5 years (10%); 5–10 years (30%); 10–20 years (40%); and more than 20 years (20%).

### GCM exercise

In Study 1, a total of 142 responses were generated by patients (n = 20) and clinicians (n = 10) to the prompt about ideal psoriasis treatment. After harmonization, 99 responses were removed primarily due to redundancy and 43 (numbered 1–43) were retained for the sorting and rating tasks (one concept, “be safe for long-term use,” was mentioned by both patients and clinicians and was inadvertently retained as a duplicate) (Table 1). Of the initial 30 participants, 27 participants completed the sorting and rating tasks (patients, n = 18; clinicians, n = 9; some participants did not sort and some completed < 75% of the tasks and were excluded) [36]. A point map generated from the sorting data through multidimensional scaling had a stress value of 0.1878, which indicated a good fit between the point map and the input similarity matrix. Seven clusters were configured from the point map and specified as domains consisting of related concepts (Fig. 1): proximal benefits, relief from symptom manifestations, efficacy/general efficacy, accessibility, safety, convenience, and administration/mode of administration. The domain labels were derived from labels participants attributed to the clusters during sorting. The average importance ratings for domains ranged from 5.92 to 9.16, represented by 1‒5 layers (representing quintiles) as shown in Fig. 1.

In Study 2, patients (N = 20) generated 42 concepts from which 37 concepts were retained after harmonization. Sorting and rating were completed by participants (n = 19; 1 patient was lost to follow-up). Cluster mapping resulted in 5 domains: efficacy/general efficacy, administration/mode of administration, application characteristics, treatment preferences, and prevention; the average importance ratings ranged from 6.85 to 8.96, represented by 1‒5 layers (representing quintiles). In both studies, patients (but not dermatologists) rated all domains > 5 in importance, indicating a higher-than-average importance for all areas of treatment represented by the domains.

### Pattern matching

In Study 1, following interpretation of the cluster maps, pattern-matching diagrams were generated to compare average domain ratings between patients and clinicians (Fig. 2). Overall, there was a high correlation in average domain ratings between patients and clinicians (Spearman r = 0.98). The domain of Accessibility was rated highest by both patients and clinicians. In addition, Safety and General Efficacy domains were also rated highly by both groups. Domains of Convenience and Mode of Administration, although rated lowest by both patients and clinicians, nonetheless had a higher absolute rating from patients than from clinicians.

### Cognitive debriefing

In Study 2, a total of 28 concepts were selected from those generated in the 2 GCM exercises for inclusion in the preliminary TAQ for the CD phase. Participants (N = 20) expressed a high level of understanding (80–100%) of all concepts. With regards to relevance, patients regarded all safety- and efficacy-related concepts > 80% for relevance; however, some concepts pertaining to administration or aesthetic issues scored lower, e.g., “Have a hands-free applicator” was considered relevant by only n = 11/20 (55%) participants. Participants chose a 4-point scale as the preferred response option for importance to the concepts, ranging from 0 = not at all important to 3 = extremely important. All participants who were asked (n = 19) stated they would be comfortable showing their clinicians their responses to the concepts and considered their responses would help them communicate with their clinician.

### Final formulation of treatment acceptability questionnaire

Of the 28 concepts cognitively debriefed, 20 were selected for inclusion in the final TAQ. Of the 8 items not included, 6 were considered the least relevant by the participants according to the CD interview (≤ 85% of participants). The other 2 items were considered possibly redundant to other included items (e.g., Item 12, “clear the skin and keep it clear” redundant to Item 1, “clear outside symptoms”). Combined with the 4-point response option, the TAQ was formulated as a verbal rating scale (Appendix 2). Based on participant suggestions from CD, a few items had wording modified from the original concept. Then, the TAQ was evaluated in an online survey of 198 patients with mild-to-severe psoriasis. For most items, a ceiling effect (≥ 25% of participants rating them 3 = extremely important) was observed; in contrast, only 1 item (Item 20, “Be an injection”) displayed a floor effect (≥ 25% of participants rating it 0 = not at all important).

### Psychometric analysis

The following psychometric analyses were conducted on the TAQ item set to inform the item performance in terms of reliability and validity of the measure.

#### Inter-item correlations

Inter-item correlations for each of the 20 items in the TAQ ranged from − 0.2 to 0.85 with the majority scoring < 0.7, indicating a substantial level of diversity among the items. Item pairs that correlated at > 0.8 are specified in Table 3 and were examined for possible redundancy. Items 2, 3, and 4 are related but nonidentical concepts of safety and effectiveness. Items 6 and 7 are also related concepts of convenience in administration. However, despite the higher correlation, the comparison of items 3 and 10 (safe for long-term use and relieve the itching) are unrelated concepts.

**Table 3:** Psychometric Analysis

Comparisons from the TAQ with Strong Correlations (≥ 0.80)		Inter-item Correlations
Item 2: Be effective in treating psoriasis for skin and other areas	Item 3: Be safe for long-term use	0.805
Item 2: Be effective in treating psoriasis for skin and other areas	Item 4: Not lose effectiveness over time	0.802
Item 3: Be safe for long term use	Item 4: Not lose effectiveness over time	0.823
Item 3: Be safe for long term use	Item 10: Relieve the itching	0.842
Item 6: Be easy to include in your (a patient’s) normal routine	Item 7: Be simple and quick to administer	0.851
Analysis of Known-Groups Validity		
Known-Group	**TAQ Total Mean (SD) Score** **(n = 197)**	***P*** **-value**
DLQI		
No or small effect on patient’s life	44.3 (12.73)	0.0072
Moderate effect on patient’s life	46.3 (8.38)	
Very large effect on patient’s life	49.2 (7.63)	
Extremely large effect on patient’s life	52.6 (6.38)	
CGI-S		
Mild	47.2 (10.65)	0.8106
Moderate	46.8 (9.12)	
Severe	47.8 (8.85)	

CGI-S - Clinician Global Impression – Severity; DLQI - Dermatology Life Quality Index; SD - standard deviation; TAQ - Treatment Acceptability Questionnaire

### Item-total correlations

Item-total correlations were above the accepted threshold of 0.4 in all cases except Item 15: Be effective using once-to-twice weekly (r = 0.346) and Item 20: Be an injection (r = 0.112). Item-pairs with correlations > 0.8 are noted in Table 3.

#### Internal consistency

The Cronbach’s alpha for the TAQ total score was 0.895 indicating very high reliability (Table 3). The alphas for the total score with the removal of each of the 20 items of the TAQ ranged from 0.884 to 0.905, suggesting that removal of any one item does not improve the overall alpha for the total score.

#### Analysis of known-groups validity

Known-groups validity specifically demonstrates the ability of the TAQ to discriminate across independent known groups (e.g., clinical severity). Table 3 presents the analysis of known-groups validity for the TAQ using the DLQI and CGI-S from n = 197 online survey patients. The TAQ total score has a range from 0 to 60, where higher scores are associated with participant’s viewing multiple treatment features and impacts as important. For the DLQI, a statistically significant monotonic relationship existed with the TAQ, where a higher mean TAQ total score was associated with increasing levels of psoriasis’ effect on a patient’s life as measured by the DLQI (i.e., impairment in QoL) (*P* = 0.0072). Thus, the higher one scores on the TAQ could be associated with a lower QoL due to psoriasis. However, no statistically significant (*P* = 0.8106) difference was observed between the patients’ rating of treatment importance (mean TAQ total score) and the clinician rating of psoriasis severity groups in the CGI-S outcomes.

#### Dimensionality

An exploratory factor analysis (EFA) was performed on the TAQ resulting in a four-factor loading for the 20 items to compare the modified instrument to the original concept domains from the GCM exercises. The proposed factor structure would consist of nine items for factor 1 (general effectiveness), three items for factor 2 (effect on hair [2 of the 3 items]), four items for factor 3 (type of medication or mode of use) and four items for factor 4 (ease of use/side effects). From a statistical perspective, Item 15 (Be effective using once to twice weekly) loaded onto factor 2, which does not conceptually (effect on hair) relate to the other two items in the factor. Similarly, Item 18 (Work internally to prevent itching) loaded onto factor 3 (type of medication or mode of use), however conceptually it did not relate to the other items within the factor. Details of the factor structure are included in Appendix 3. The clustering of the four factors aligns to the initial GCM domains from both study samples with concepts such as general efficacy, convenience and ease of administration, and safety and longterm use.

Additionally, a confirmatory factor analysis (CFA) was conducted to test the dimensionality of the TAQ with maximum likelihood estimation. A one-factor solution showed good fit (RMSEA = 0.1283; Table 2, Appendix 4). The standardized factor loadings were acceptable (> 0.5; Table 1, Appendix 4), however, four items (9, 15, 17, and 20) had low factor loadings.

## Discussion

This study was undertaken to breach the information gap regarding patient expectations of a psoriasis treatment that addresses their needs. A primary objective of these studies was to develop an instrument that provides patient preferences of treatment features that would address unmet needs from psoriasis patients across the severity spectrum. The TAQ was created to capture patient expectations and preferences for psoriasis treatment and was found to be valid and reliable through psychometric evaluations.

Two mixed-methods studies were conducted in our studies, including qualitative inquiry using GCM methods and telephone interviews as well as online quantitative surveys and psychometric analyses. In these studies, a de novo treatment acceptability measure was envisioned from concepts derived from Study 1 in moderate-to-severe psoriasis patients; formalized in Study 2 as the inchoate measure was formalized in Study 2 through additional concepts obtained from mild psoriasis patients as well as CD interviews and psychometric analyses. In contrast to instruments that measure burden/impact of psoriasis (like the DLQI) or treatment effectiveness, the newly developed 20-item TAQ informs patient expectations of and preference for psoriasis treatment. In contrast, DLQI domains cover symptoms, daily activities, and personal relationships (to name a few), and while one question asks if treatments have been a problem for the patient, the instrument does not inform preferences for specific selection of treatment attributes or preferences. Availability of multiple administration modes, different tolerability profiles, and variations in efficacy and duration have increased the need for a stronger understanding of patient acceptability and preference of treatments, and studies have been conducted in this area [42, 43] Indeed, shared decision making is being embraced by both healthcare providers and patients [44].

Another outcome of note was that while patients and clinicians agreed on Safety, Accessibility, and General Efficacy domains as of prime importance, the domains of Convenience and Mode of Administration received higher value from patients than from clinicians. This suggests that clinicians may be more focused on treatment efficacy and safety than on personal and lifestyle needs of the patient. These factors have been shown to influence treatment acceptance, compliance, and continuation [45–47]; thus, a better awareness of the part of clinicians on patient convenience and administration preference may be warranted.

Although analysis of the TAQ revealed a four-factor structure, suggesting a multidimensional measure, results from the CFA indicated support for a unidimensional measure with the potential for a second factor. An overall score was applied to assess item performance of the measure in this paper since the CFA supported unidimensionality, however, further validation within real-world clinical studies should assess the dimensionality, domain scoring and overall scores with additional samples.

The studies have some limitations. Because GCM is an online exercise, patients or clinicians who are not comfortable with computer use or do not have access to the internet were unlikely to participate. The demographic population of these studies was predominantly White/Caucasian with little variability, indicating additional research in other racial/ethnic populations is needed.

Due to the subjective nature of patient-focused research, there may be discordance between the patients and clinicians. For instance, in Study 2, safety and efficacy concepts were included in one domain, whereas they were in separate domains in Study 1. Results from the EFA suggest a four-factor solution, whereas the CFA results supported a unidimensional model. Both structures still have concordance with the GCM derived domains and support the patient-generated concepts as a whole.

Nonetheless, these findings provide evidence that the TAQ can be a valuable instrument to direct clinicians to customize treatments that best align with what is important for each patient for the treatment of their psoriasis. There is potential for use in real-world clinical studies with further validation and to contribute to fulfilling unmet needs of psoriasis management.

## Conclusions

The TAQ is a novel patient-reported instrument developed to inform healthcare providers of patients’ expectations and preferences with regards to their psoriasis treatment and help healthcare providers in managing psoriasis based on patient preference and tolerability. Further, the TAQ may facilitate better communication and shared decision making between patients and physicians.

### Electronic supplementary material

Below is the link to the electronic supplementary material.

Supplementary Material 1

Supplementary Material 2

Supplementary Material 3

Supplementary Material 4

## Data Availability

The datasets generated and/or analyzed during the current study are not publicly available but are available from the corresponding author on reasonable request.
