# Supplementary material for: Development and validation of a psoriasis treatment acceptability measure through group concept mapping
Source: Health Qual Life Outcomes. 2023 Aug 8;21:83. doi: 10.1186/s12955-023-02162-6 (PMC10408213; doi:10.1186/s12955-023-02162-6)

**Psoriasis Manuscript Appendix 1**

**Figure 1S. Schematic of GCM Exercise**

Site identification and participant recruitment

Identification of key concepts


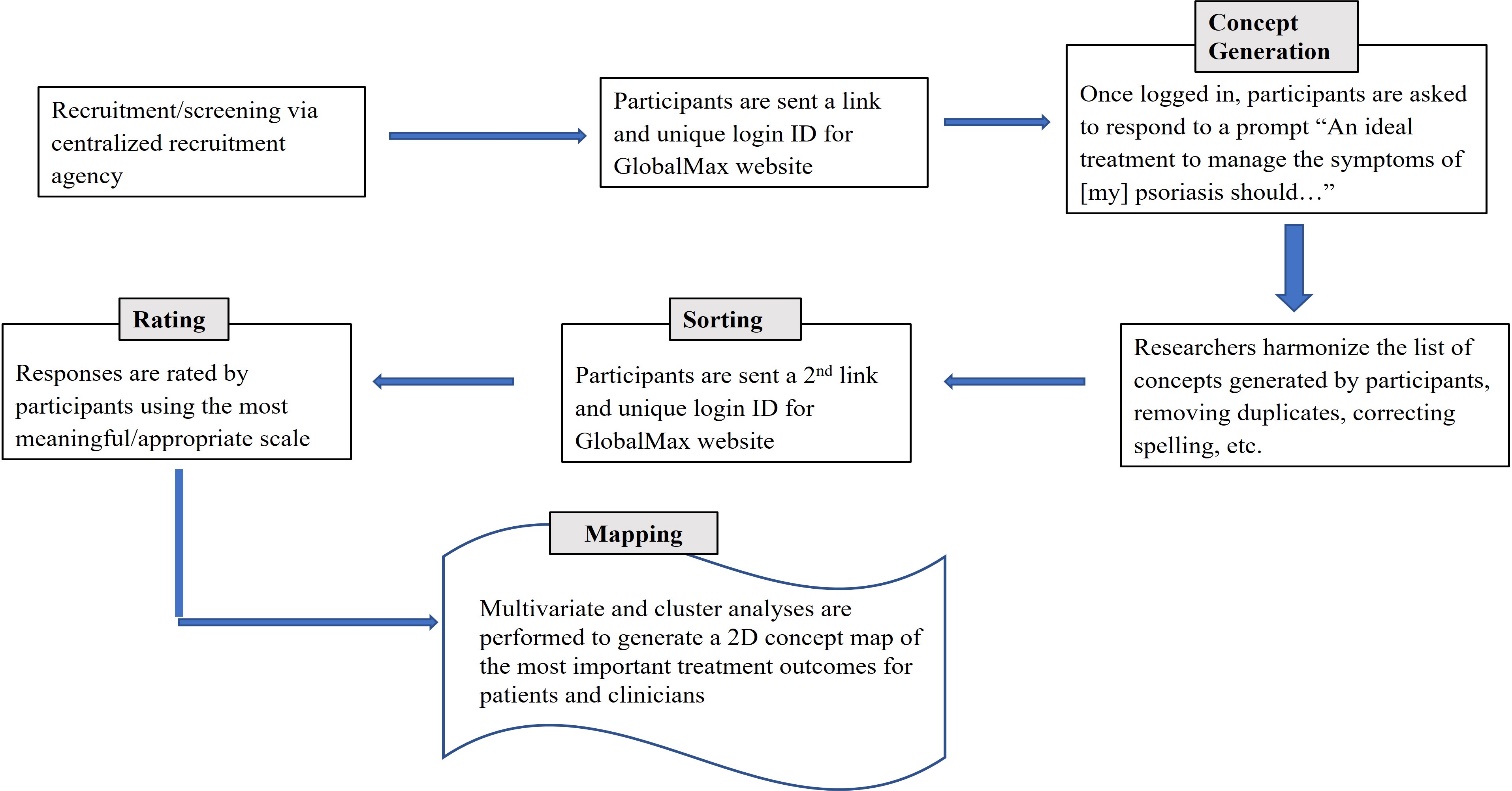


**Figure 2S. Flowchart of Studies 1 and 2**


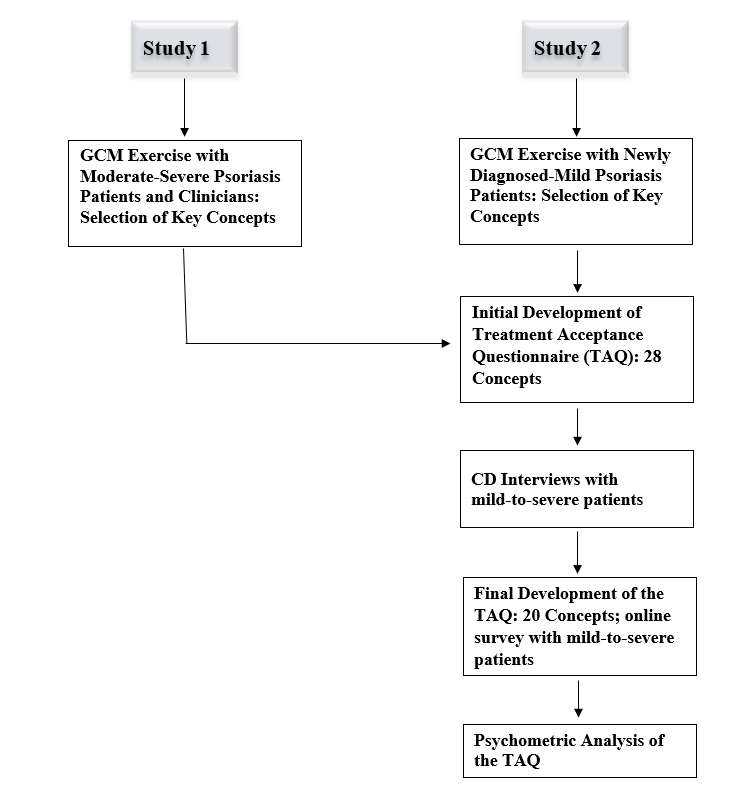

Supplement: Supplementary file 1 — Supplementary Material 1 [file 12955_2023_2162_MOESM1_ESM.docx]
