# Supplementary material for: Development and validation of a psoriasis treatment acceptability measure through group concept mapping
Source: Health Qual Life Outcomes. 2023 Aug 8;21:83. doi: 10.1186/s12955-023-02162-6 (PMC10408213; doi:10.1186/s12955-023-02162-6)
Supplement: Supplementary file 2 — Supplementary Material 2 [file 12955_2023_2162_MOESM2_ESM.docx]

**Psoriasis Manuscript Appendix 2**

**Treatment Acceptability Questionnaire (TAQ)**

Instructions:

The following statements describe features that an ideal treatment would have. Please rate the statements based on **importance** for the treatment of **your** psoriasis.

Response Options:

o Not at all important (0)

o Somewhat important (1)

o Moderately important (2)

o Extremely important (3)

| Statement | Item Number | GCM Source |
| --- | --- | --- |
| Clear symptoms that are visible* | TAQ1 | Study 1 |
| Be effective in treating psoriasis for skin and other areas* | TAQ2 | Study 1 |
| Be safe for long term use | TAQ3 | Study 1 |
| Not lose effectiveness over time* | TAQ4 | Study 1 |
| Safe to take with other medications* | TAQ5 | Study 1 |
| Be easy to include in your (a patient's) normal routine* | TAQ6 | Study 1 |
| Be simple and quick to administer | TAQ7 | Study 1 |
| Be painless to use | TAQ8 | Study 1 |
| Have an alternate to injections | TAQ9 | Study 1 |
| Relieve the itching* | TAQ10 | Study 2 |
| Have little to no side effects | TAQ11 | Study 2 |
| Prevent symptoms from coming back* | TAQ12 | Study 2 |
| Be easy to apply to all areas which need treatment | TAQ13 | Study 2 |
| Prevent psoriasis build-up on my scalp* | TAQ14 | Study 2 |
| Be effective using once to twice weekly | TAQ15 | Study 2 |
| Absorb invisibly in my skin | TAQ16 | Study 2 |
| Be gentle enough to use daily without damaging my hair | TAQ17 | Study 2 |
| Work internally to prevent itching* | TAQ18 | Study 2 |
| Be a pill for long term use | TAQ19 | Study 2 |
| Be an injection* | TAQ20 | Study 1 |

*Wording modified from original statement based on survey feedback
