# Supplementary material for: Development and validation of a psoriasis treatment acceptability measure through group concept mapping
Source: Health Qual Life Outcomes. 2023 Aug 8;21:83. doi: 10.1186/s12955-023-02162-6 (PMC10408213; doi:10.1186/s12955-023-02162-6)
Supplement: Supplementary file 3 — Supplementary Material 3 [file 12955_2023_2162_MOESM3_ESM.docx]

**Psoriasis Manuscript Appendix 3**

## Exploratory Factor Analysis

The figure below presents the initial scree plot and percentage of variance explained for the TAQ. Four factors were considered based on Kaiser’s criterion (eigenvalue ≥ 1.0). Sixty-two percent of the variance can be explained using four factors. Full outputs on determining the number of factors can be found on the following page.

Appendix 3 Figure 1: Initial Scree Plot and Percentage of Variance Explained for the TAQ

The table below presents the factor loading for the TAQ using a four-factor solution using varimax and promax rotations. The analyses showed that 15 out of the 20 items in the TAQ loaded (a loading ≥0.40) with no cross-loadings between factors and agreement between the varimax rotation and promax rotation methodologies. Using the varimax rotation, there were cross-loadings between two factors for item 7 and item 18. However, with the promax rotation, these two items loaded on a single factor. Therefore, it was decided that item 7 would be loaded onto factor 4 and item 18 would be loaded to factor 3, as these were the highest correlations for both the varimax and promax rotations. Item 13 and item 16 did not load using the promax rotation but loaded with the varimax rotation. Therefore, these items were included in their respective factors using the varimax rotation.

The proposed factor structure consists of nine items for factor 1 (general effectiveness), three items for factor 2 (effect on hair [2 of the 3 items]), four items for factor 3 (type of medication or mode of use) and four items for factor 4 (ease of use/side effects). From a statistical prospective, item 15 (Be effective using once to twice weekly) loaded onto factor 2, which does not conceptually (effect on hair) relate to the other two items in the factor. Similarly, item 18 (Work internally to prevent itching) loaded onto factor 3 (type of medication or mode of use), however conceptually it does not relate with the other items within the factor.

Table Appendix 3.1 Four-Factor Solution for TAQ

|  |  | Rotated Varimax Factor Loadings (top number)  Rotated Promax Factor Pattern (lower number) | | | |
| --- | --- | --- | --- | --- | --- |
| Item | Label | Factor 1 | Factor 2 | Factor 3 | Factor 4 |
| Item 1 | Clear symptoms that are visible | 0.70679*  0.79618* |  |  |  |
| Item 2 | Be effective in treating psoriasis for skin and other areas | 0.79555*  0.87073* |  |  |  |
| Item 3 | Be safe for long term use | 0.78168*  0.80547* |  |  |  |
| Item 4 | Not lose effectiveness over time | 0.81828*  0.86382* |  |  |  |
| Item 5 | Safe to take with other medications | 0.67104*  0.66856* |  |  |  |
| Item 6 | Be easy to include in your (a patient's) normal routine | 0.56069*  0.47955* |  |  |  |
| Item 7 | Be simple and quick to administer | 0.41285*  0.25762* |  |  | 0.56935*  0.48206* |
| Item 8 | Be painless to use |  |  |  | 0.66475*  0.62488* |
| Item 9 | Have an alternate to injections |  |  |  | 0.82362*  0.88196* |
| Item 10 | Relieve the itching | 0.68287*  0.67460* |  |  |  |
| Item 11 | Have little to no side effects |  |  |  | 0.51965*  0.42780* |
| Item 12 | Prevent symptoms from coming back | 0.57433*  0.51105* |  |  |  |
| Item 13 | Be easy to apply to all areas which need treatment | 0.41924*  0.28510 |  |  |  |
| Item 14 | Prevent psoriasis build-up on my scalp |  | 0.81403*  0.86810* |  |  |
| Item 15 | Be effective using once to twice weekly |  | 0.67635*  0.69136* |  |  |
| Item 16 | Absorb invisibly in my skin |  |  | 0.43732*  0.38200 |  |
| Item 17 | Be gentle enough to use daily without damaging my hair |  | 0.73078*  0.78331* |  |  |
| Item 18 | Work internally to prevent itching |  | 0.41436*  0.33916* | 0.62474*  0.60822* |  |
| Item 19 | Be a pill for long term use |  |  | 0.72557*  0.78907* |  |
| Item 20 | Be an injection |  |  | 0.62124*  0.73327* |  |

Abbreviation: TAQ = Treatment Acceptability Questionnaire.

* Indicates a loading ≥ 0.40 (standard threshold)
