# Supplementary material for: Development and validation of a psoriasis treatment acceptability measure through group concept mapping
Source: Health Qual Life Outcomes. 2023 Aug 8;21:83. doi: 10.1186/s12955-023-02162-6 (PMC10408213; doi:10.1186/s12955-023-02162-6)
Supplement: Supplementary file 4 — Supplementary Material 4 [file 12955_2023_2162_MOESM4_ESM.rtf]

Table 1. Model-fit results of the confirmatory factor analysis of the TAQ (20 items) using WLSMV1,2

Model	×2 value	Df	P value	RMSEA [90% CI]	
Unidimensional model with 20 items	718.7294	170	<.0001	0.1283 [0.1187; 0.1381]	
1-	WLSMV; robust weighted least squares
2-	Sample size for the model was n=xxx.

Table 2. Factor loadings for the TAQ CFA

Item	Factor Loading	Residual Variance	
TAQ1 Clear symptoms that are visible	0.487	0.763	
TAQ2 Be effective in treating psoriasis for skin and other areas	0.623	0.612	
TAQ3 Be safe for long term use	0.741	0.451	
TAQ4 Not lose effectiveness over time	0.728	0.471	
TAQ5 Safe to take with other medications	0.696	0.516	
TAQ6 Be easy to include in your (a patient's) normal routine	0.754	0.432	
TAQ7 Be simple and quick to administer	0.749	0.438	
TAQ8 Be painless to use	0.645	0.584	
TAQ9 Have an alternate to injections	0.400	0.840	
TAQ10 Relieve the itching	0.716	0.488	
TAQ11 Have little to no side effects	0.723	0.477	
TAQ12 Prevent symptoms from coming back	0.731	0.466	
TAQ13 Be easy to apply to all areas which need treatment	0.721	0.481	
TAQ14 Prevent psoriasis build-up on my scalp	0.461	0.788	
TAQ15 Be effective using once to twice weekly	0.288	0.917	
TAQ16 Absorb invisibly in my skin	0.535	0.714	
TAQ17 Be gentle enough to use daily without damaging my hair	0.386	0.851	
TAQ18 Work internally to prevent itching	0.581	0.663	
TAQ19 Be a pill for long term use	0.474	0.776	
TAQ20 Be an injection	0.036	0.999	


Fit Summary	Interpretation	
Absolute Index	Fit Function	3.6670		
	Chi-Square	718.7294		
	Chi-Square DF	170		
	Pr > Chi-Square	<.0001	Less than 0.05 (the hypothesis of perfect fit cannot be rejected)	
	Root Mean Square Residual (RMR)	0.0685	Less than the < 0.08 criteria	
	Standardized RMR (SRMR)	0.0938	Slightly higher than < 0.08 criteria	
	Goodness of Fit Index (GFI)	0.6994	Less than the expected > 0.95 criteria	
Parsimony Index	Adjusted GFI (AGFI)	0.6286	Less than the expected > 0.90 criteria	
	Parsimonious GFI	0.6258	Less than the expected > 0.90 criteria	
	RMSEA Estimate	0.1283	Slightly higher than < 0.08 criteria	
	RMSEA Lower 90% Confidence Limit	0.1187		
	RMSEA Upper 90% Confidence Limit	0.1381		
	Probability of Close Fit	<.0001		

Factor Loading Matrix: Estimate/StdErr/t-value/p-value	
	TAQ_FACTOR	
_TAQ1	0.4356
0.0619
7.0372
<.0001
[_Parm01]	
_TAQ2	0.4936
0.0523
9.4369
<.0001
[_Parm02]	
_TAQ3	0.5127
0.0432
11.8715
<.0001
[_Parm03]	
_TAQ4	0.5356
0.0463
11.5780
<.0001
[_Parm04]	
_TAQ5	0.5509
0.0506
10.8893
<.0001
[_Parm05]	
_TAQ6	0.5680
0.0467
12.1693
<.0001
[_Parm06]	
_TAQ7	0.6283
0.0521
12.0712
<.0001
[_Parm07]	
_TAQ8	0.5026
0.0510
9.8650
<.0001
[_Parm08]	
_TAQ9	0.4287
0.0758
5.6525
<.0001
[_Parm09]	
_TAQ10	0.4065
0.0359
11.3155
<.0001
[_Parm10]	
_TAQ11	0.5968
0.0520
11.4785
<.0001
[_Parm11]	
_TAQ12	0.4456
0.0383
11.6417
<.0001
[_Parm12]	
_TAQ13	0.4676
0.0409
11.4283
<.0001
[_Parm13]	
_TAQ14	0.3723
0.0563
6.6075
<.0001
[_Parm14]	
_TAQ15	0.2647
0.0665
3.9821
<.0001
[_Parm15]	
_TAQ16	0.4022
0.0513
7.8421
<.0001
[_Parm16]	
_TAQ17	0.3662
0.0674
5.4304
<.0001
[_Parm17]	
_TAQ18	0.4290
0.0496
8.6559
<.0001
[_Parm18]	
_TAQ19	0.5133
0.0753
6.8201
<.0001
[_Parm19]	
_TAQ20	0.0358
0.0727
0.4917
0.6229
[_Parm20]	


Factor Covariance Matrix: Estimate/StdErr/t-value/p-value	
	TAQ_FACTOR	
TAQ_FACTOR	1.0000


	


Error Variances	
Variable	Parameter	Estimate	Standard
Error	t Value	Pr > |t|	
_TAQ1	_Add01	0.61041	0.06308	9.6768	<.0001	
_TAQ2	_Add02	0.38445	0.04070	9.4452	<.0001	
_TAQ3	_Add03	0.21614	0.02395	9.0260	<.0001	
_TAQ4	_Add04	0.25493	0.02804	9.0917	<.0001	
_TAQ5	_Add05	0.32384	0.03510	9.2269	<.0001	
_TAQ6	_Add06	0.24498	0.02736	8.9536	<.0001	
_TAQ7	_Add07	0.30775	0.03428	8.9781	<.0001	
_TAQ8	_Add08	0.35468	0.03778	9.3886	<.0001	
_TAQ9	_Add09	0.96422	0.09876	9.7630	<.0001	
_TAQ10	_Add10	0.15748	0.01722	9.1462	<.0001	
_TAQ11	_Add11	0.32503	0.03567	9.1128	<.0001	
_TAQ12	_Add12	0.17351	0.01911	9.0779	<.0001	
_TAQ13	_Add13	0.20221	0.02216	9.1232	<.0001	
_TAQ14	_Add14	0.51461	0.05302	9.7066	<.0001	
_TAQ15	_Add15	0.77454	0.07876	9.8347	<.0001	
_TAQ16	_Add16	0.40408	0.04204	9.6126	<.0001	
_TAQ17	_Add17	0.76764	0.07854	9.7744	<.0001	
_TAQ18	_Add18	0.36170	0.03793	9.5348	<.0001	
_TAQ19	_Add19	0.91067	0.09396	9.6922	<.0001	
_TAQ20	_Add20	0.96614	0.09760	9.8985	<.0001	


Squared Multiple Correlations	
Variable	Error Variance	Total Variance	R-Square	
_TAQ1	0.61041	0.80017	0.2371	
_TAQ2	0.38445	0.62804	0.3879	
_TAQ3	0.21614	0.47902	0.5488	
_TAQ4	0.25493	0.54175	0.5294	
_TAQ5	0.32384	0.62737	0.4838	
_TAQ6	0.24498	0.56760	0.5684	
_TAQ7	0.30775	0.70253	0.5619	
_TAQ8	0.35468	0.60732	0.4160	
_TAQ9	0.96422	1.14799	0.1601	
_TAQ10	0.15748	0.32275	0.5121	
_TAQ11	0.32503	0.68119	0.5228	
_TAQ12	0.17351	0.37206	0.5336	
_TAQ13	0.20221	0.42085	0.5195	
_TAQ14	0.51461	0.65322	0.2122	
_TAQ15	0.77454	0.84461	0.0830	
_TAQ16	0.40408	0.56583	0.2859	
_TAQ17	0.76764	0.90174	0.1487	
_TAQ18	0.36170	0.54574	0.3372	
_TAQ19	0.91067	1.17419	0.2244	
_TAQ20	0.96614	0.96742	0.00132	


Factor Scores Regression Coefficients	
	TAQ_FACTOR	
_TAQ1	0.0481	
_TAQ2	0.0866	
_TAQ3	0.1600	
_TAQ4	0.1416	
_TAQ5	0.1147	
_TAQ6	0.1563	
_TAQ7	0.1377	
_TAQ8	0.0956	
_TAQ9	0.0300	
_TAQ10	0.1741	
_TAQ11	0.1238	
_TAQ12	0.1732	
_TAQ13	0.1559	
_TAQ14	0.0488	
_TAQ15	0.0230	
_TAQ16	0.0671	
_TAQ17	0.0322	
_TAQ18	0.0800	
_TAQ19	0.0380	
_TAQ20	0.002496	

Standardized Factor Loading Matrix: Estimate/StdErr/t-value/p-value	
	TAQ_FACTOR	
_TAQ1	0.4870
0.0571
8.5281
<.0001
[_Parm01]	
_TAQ2	0.6228
0.0464
13.4172
<.0001
[_Parm02]	
_TAQ3	0.7408
0.0351
21.1169
<.0001
[_Parm03]	
_TAQ4	0.7276
0.0364
19.9681
<.0001
[_Parm04]	
_TAQ5	0.6956
0.0396
17.5447
<.0001
[_Parm05]	
_TAQ6	0.7539
0.0337
22.3637
<.0001
[_Parm06]	
_TAQ7	0.7496
0.0342
21.9437
<.0001
[_Parm07]	
_TAQ8	0.6450
0.0444
14.5173
<.0001
[_Parm08]	
_TAQ9	0.4001
0.0626
6.3937
<.0001
[_Parm09]	
_TAQ10	0.7156
0.0377
19.0019
<.0001
[_Parm10]	
_TAQ11	0.7231
0.0369
19.5953
<.0001
[_Parm11]	
_TAQ12	0.7305
0.0361
20.2112
<.0001
[_Parm12]	
_TAQ13	0.7208
0.0371
19.4104
<.0001
[_Parm13]	
_TAQ14	0.4606
0.0589
7.8243
<.0001
[_Parm14]	
_TAQ15	0.2880
0.0681
4.2320
<.0001
[_Parm15]	
_TAQ16	0.5347
0.0536
9.9672
<.0001
[_Parm16]	
_TAQ17	0.3856
0.0634
6.0840
<.0001
[_Parm17]	
_TAQ18	0.5807
0.0500
11.6137
<.0001
[_Parm18]	
_TAQ19	0.4737
0.0580
8.1673
<.0001
[_Parm19]	
_TAQ20	0.0364
0.0739
0.4921
0.6226
[_Parm20]	


Factor Correlation Matrix: Estimate/StdErr/t-value/p-value	
	TAQ_FACTOR	
TAQ_FACTOR	1.0000


	


Standardized Results for Error Variances	
Variable	Parameter	Estimate	Standard
Error	t Value	Pr > |t|	
_TAQ1	_Add01	0.76286	0.05561	13.7171	<.0001	
_TAQ2	_Add02	0.61213	0.05782	10.5875	<.0001	
_TAQ3	_Add03	0.45120	0.05198	8.6807	<.0001	
_TAQ4	_Add04	0.47057	0.05303	8.8741	<.0001	
_TAQ5	_Add05	0.51619	0.05515	9.3594	<.0001	
_TAQ6	_Add06	0.43162	0.05083	8.4912	<.0001	
_TAQ7	_Add07	0.43806	0.05122	8.5529	<.0001	
_TAQ8	_Add08	0.58400	0.05731	10.1900	<.0001	
_TAQ9	_Add09	0.83992	0.05007	16.7737	<.0001	
_TAQ10	_Add10	0.48794	0.05390	9.0534	<.0001	
_TAQ11	_Add11	0.47715	0.05336	8.9413	<.0001	
_TAQ12	_Add12	0.46636	0.05281	8.8315	<.0001	
_TAQ13	_Add13	0.48047	0.05353	8.9756	<.0001	
_TAQ14	_Add14	0.78781	0.05424	14.5246	<.0001	
_TAQ15	_Add15	0.91704	0.03921	23.3890	<.0001	
_TAQ16	_Add16	0.71413	0.05736	12.4493	<.0001	
_TAQ17	_Add17	0.85129	0.04889	17.4135	<.0001	
_TAQ18	_Add18	0.66277	0.05807	11.4126	<.0001	
_TAQ19	_Add19	0.77557	0.05496	14.1120	<.0001	
_TAQ20	_Add20	0.99868	0.00537	185.9	<.0001	

Stepwise Multivariate Wald Test	
Parm	Cumulative Statistics	Univariate Increment	
	Chi-Square	DF	Pr > ChiSq	Chi-Square	Pr > ChiSq	
_Parm20	0.24175	1	0.6229	0.24175	0.6229	

Note:	There is no parameter to free in the default LM tests for the factor loadings. Ranking is not displayed.	

Note:	No LM statistic in the default test set for the covariances of factors is nonsingular. Ranking is not displayed.	


Rank Order of the 10 Largest LM Stat for Error Variances and Covariances	
Error
of	Error
of	LM Stat	Pr > ChiSq	Parm
Change	
_TAQ17	_TAQ14	52.90429	<.0001	0.33200	
_TAQ2	_TAQ1	40.15626	<.0001	0.22744	
_TAQ4	_TAQ2	36.24126	<.0001	0.14476	
_TAQ7	_TAQ6	35.31786	<.0001	0.13087	
_TAQ15	_TAQ1	24.87960	<.0001	-0.24865	
_TAQ3	_TAQ2	23.82820	<.0001	0.10853	
_TAQ16	_TAQ15	22.56923	<.0001	0.19334	
_TAQ15	_TAQ14	22.54824	<.0001	0.21701	
_TAQ4	_TAQ3	22.37663	<.0001	0.08783	
_TAQ10	_TAQ3	21.01611	<.0001	0.06664	
